# Supplementary material for: The Roots of Rye (Secale cereale L.) Are Capable of Synthesizing Benzoxazinoids
Source: Int J Mol Sci. 2021 Apr 28;22(9):4656. doi: 10.3390/ijms22094656 (PMC8124178; doi:10.3390/ijms22094656)
Supplement: Supplementary file 1 [file ijms-22-04656-s001.zip › ijms-1173605-supplementary.pdf]

The roots of rye (*Secale cereale* L.) are capable of synthesizing benzoxazinoids

Monika Rakoczy-Trojanowska[1,*], Bartosz M. Szabała[1], Elżbieta Różańska[2], Mariusz Kowalczyk[3], Wojciech Burza[1], Beata Bakera[1], Magdalena Święcicka[1,2]

[1] Department of Plant Genetics, Breeding and Biotechnology, Institute of Biology, Warsaw University of Life Sciences (SGGW), Nowoursynowska 166 St., 02-787 Warsaw, Poland

[2] Department of Botany, Institute of Biology, Warsaw University of Life Sciences (SGGW), Nowoursynowska 166 St., 02-787 Warsaw, Poland

[3] Department of Biochemistry and Crop Quality, Institute of Soil Science and Plant Cultivation - State Research Institute, Czartoryskich 8 St., 24-100 Puławy, Poland

*corresponding author: monika_rakoczy_trojanowska@sggw.edu.pl tel. +48-225932150

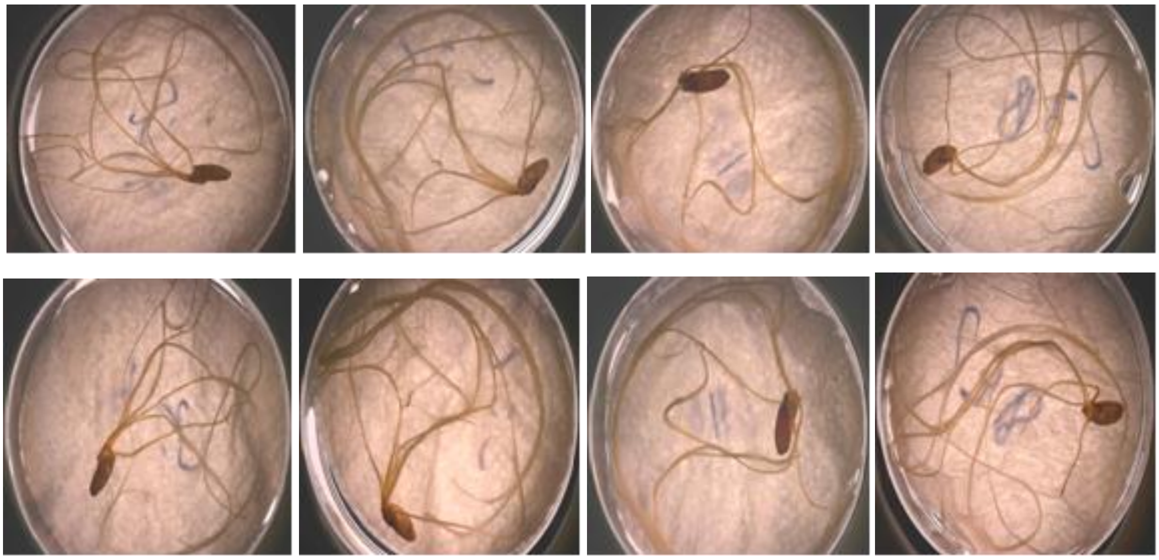

Figure S1. Twelve-day-old roots developed from seeds deprived of coleoptile of rye inbred line L318.

Table S1. Relative transcript levels of *ScBx1* and *ScIgl* in rye RDC, RIV, R2W, and L2W.

Analyzed organ/organoid	Genes	
	ScBx1	*ScIgl*
RDC	0.0014 (0.0002)	0.0224 (0.0100)
RIV	0,0002 (0.0000)	nd (-)
KI R2W	0.0028 (0.0004)	0.0090 (0.0010)
KII L2W	0.0697 (0.0067)	0.0020 (0.0010)

RDC - roots developed from seeds deprived of the coleoptile at 2 days after sowing
RIV - roots developed *in vitro*
KI R2W - control reference organ: roots from 2-week-old seedlings
KII L2W - control reference organ: leaves from 2-week-old seedlings
nd – not detected
Values in brackets are standard errors.

The same methodology was used to analyse *ScIgl* and *ScBx1* transcript levels (described in the main text). The sequences of primers used in PCR were as follows:

F: AACACCAGCTACACCATCAGAG

R: GTGGGTTTACAGTCGCCCTA

Table S2. Contents of six benzoxazinoids in RDC, RIV, R2W, and L2W of rye inbred line L318.

Analyzed organ/organoid	BX [µg/mg d.m.]					
	HBOA	GDIBOA	DIBOA	GDIMBOA	DIMBOA	MBOA
RDC	0.028	0.070	0.248	0.772	1.194	0.410
	(0.001)	(0.000)	(0.003)	(0.001)	(0.008)	(0.021)
RIV	0.004	0.010	0.003	nd	0.010	0.001
	(0.000)	(0.001)	(0.000)	-	(0.001)	(0.000)
KI R2W	0.018	0.318	0.114	8.073	0.536	0.427
	(0.000)	(0.013)	(0.006)	(0.000)	(0.013)	(0.013)
KII L2W	0.202	2.507	4.475	0.230	0.013	0.044
	(0.090)	(0.726)	(1.405)	(0.128)	(0.010)	(0.015)

RDC - roots developed from seeds deprived of the coleoptile at 2 days after sowing
RIV - roots developed *in vitro*
KI R2W - control reference organ: roots from 2-week-old seedlings
KII L2W - control reference organ: leaves from 2-week-old seedlings
nd – not detected
Values in brackets are standard errors.

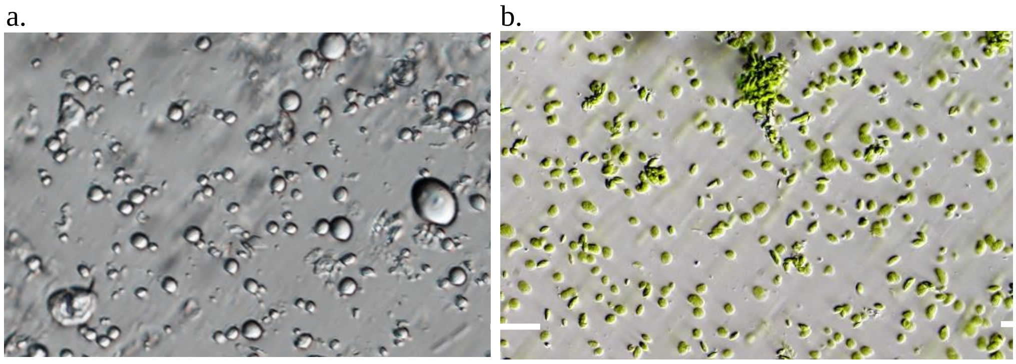

Figure S2. Plastids isolated from roots developed from (a) seeds deprived of coleoptile at 2 days after sowing and (b) leaves of 2-week-old seedlings of rye inbred line L318. Scale bars = 50 µm.

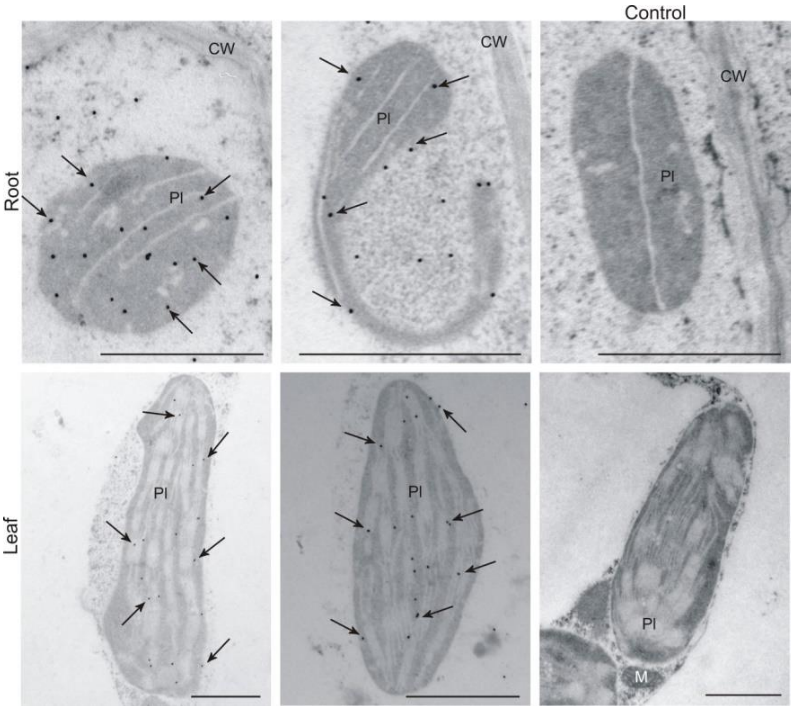

Figure S3. Localisation of indole-3-glycerol phosphate lyase by immunogold labelling and transmission microscopy in roots (R2W) and leaves (L2W) of 2-week-old seedlings of rye inbred line L318. Abbreviations: CW- cell wall, M- mitochondrion, Pl- plastid. Scale bars = 1 µm.
